# Identification of necroptosis-related features in diabetic nephropathy and analysis of their immune microenvironent and inflammatory response

**DOI:** 10.3389/fcell.2023.1271145

**Published:** 2023-11-07

**Authors:** Kaibo Hu, Ruifeng He, Minxuan Xu, Deju Zhang, Guangyu Han, Shengye Han, Leyang Xiao, Panpan Xia, Jitao Ling, Tingyu Wu, Fei Li, Yunfeng Sheng, Jing Zhang, Peng Yu

**Affiliations:** ^1^ Department of Endocrinology and Metabolism, The Second Affiliated Hospital of Nanchang University, Nanchang, China; ^2^ The Second Clinical Medical College, Nanchang University, Nanchang, China; ^3^ Branch of National Clinical Research Center for Metabolic Diseases, Nanchang, China; ^4^ Institute for the Study of Endocrinology and Metabolism in Jiangxi Province, Nanchang, China; ^5^ Food and Nutritional Sciences, School of Biological Sciences, The University of Hong Kong, Pokfulam, Hong Kong SAR, China; ^6^ Department of Anesthesiology, The Second Affiliated Hospital of Nanchang University, Nanchang, China

**Keywords:** diabetic nephropathy, necroptosis, immune landscape, inflammatory response, diagnostic model

## Abstract

**Background:** Diabetic nephropathy (DN) was considered a severe microvascular complication of diabetes, which was recognized as the second leading cause of end-stage renal diseases. Therefore, identifying several effective biomarkers and models to diagnosis and subtype DN is imminent. Necroptosis, a distinct form of programmed cell death, has been established to play a critical role in various inflammatory diseases. Herein, we described the novel landscape of necroptosis in DN and exploit a powerful necroptosis-mediated model for the diagnosis of DN.

**Methods:** We obtained three datasets (GSE96804, GSE30122, and GSE30528) from the Gene Expression Omnibus (GEO) database and necroptosis-related genes (NRGs) from the GeneCards website. Via differential expression analysis and machine learning, significant NRGs were identified. And different necroptosis-related DN subtypes were divided using consensus cluster analysis. The principal component analysis (PCA) algorithm was utilized to calculate the necroptosis score. Finally, the logistic multivariate analysis were performed to construct the necroptosis-mediated diagnostic model for DN.

**Results:** According to several public transcriptomic datasets in GEO, we obtained eight significant necroptosis-related regulators in the occurrence and progress of DN, including CFLAR, FMR1, GSDMD, IKBKB, MAP3K7, NFKBIA, PTGES3, and SFTPA1 via diversified machine learning methods. Subsequently, employing consensus cluster analysis and PCA algorithm, the DN samples in our training set were stratified into two diverse necroptosis-related subtypes based on our eight regulators’ expression levels. These subtypes exhibited varying necroptosis scores. Then, we used various functional enrichment analysis and immune infiltration analysis to explore the biological background, immune landscape and inflammatory status of the above subtypes. Finally, a necroptosis-mediated diagnostic model was exploited based on the two subtypes and validated in several external verification datasets. Moreover, the expression level of our eight regulators were verified in the singe-cell level and glomerulus samples. And we further explored the relationship between the expression of eight regulators and the kidney function of DN.

**Conclusion:** In summary, our necroptosis scoring model and necroptosis-mediated diagnostic model fill in the blank of the relationship between necroptosis and DN in the field of bioinformatics, which may provide novel diagnostic insights and therapy strategies for DN.

## Introduction

Diabetic nephropathy (DN) as an important microvascular complication in diabetes patients, which was strongly associated with the development of end-stage renal diseases (Papadopoulou-Marketou et al., 2018; Xiong and Zhou, 2019; Sasso et al., 2021). The lesion of DN is mainly located in the glomerulus. There was a noticeable accumulation of extracellular matrix within the glomerulus and tubulointerstitial compartments in DN. This accumulation is often accompanied by the thickening and hyalinization of the renal vascular system (Kanwar et al., 2011). Damage to renal blood vessels can lead to incomplete blood filtration, leading to protein infiltration into urine. Therefore, when continuous microalbuminuria occurs in patients with diabetes, it can be suspected that DN exist (Gross et al., 2005; Papadopoulou-Marketou et al., 2017; Vijay et al., 2018). Importantly, DN is related to the occurrence of various diseases including renal failure, cardiovascular diseases like stroke and hypertension, cerebral vascular disease like cerebral hemorrhage and cerebral embolism, digestive system diseases and musculoskeletal disorders. Because of this, it was an enormous threat to human health and aggravates expenditure of public health finance (Flyvbjerg, 2017). Moreover, hyperglycemia plays a pivotal role in the progression of DN by contributing to various mechanisms. These include increased oxidative stress, formation of renal polyols, activation of protein kinase C-mitogen-activated protein kinases (PKC-MAPK), accumulation of advanced glycation end products, systemic hypertension, and elevated intraglomerular pressure. All these factors collectively contribute significantly to the occurrence, development, and deterioration of DN. (Kikkawa et al., 2003). Clinically, people commonly used microalbuminuria to evaluate the progress of DN in the past. However, it is not accurate to assesse the severity or prognosis solely based on the degree of proteinuria, because not all diabetes patients with renal failure experience significant albuminuria (Qi et al., 2017). Besides, the other two broad study outcome measures, hard renal end-points (e.g., death, end stage renal disease, chronic kidney disease) and the rate of GFR/eGFR (estimated glomerular filtration rate) decline all have certain defects. Studies using hard renal end‐points require large sample sizes to reach statistical significance. The creatinine levels and derived estimates of GFR are less precise (Macis et al., 2014; Radcliffe et al., 2017). Therefore, it is necessary to find new markers or models for DN, which can accurately assess the progression of DN to provide assistance for more effective treatment.

Throughout the whole life, cell death serves an indispensable function. Traditionally, cell death is merely categorized into programmed cell death (PCD) and accidental cell death (ACD). Nowadays, PCD accounts for the majority of cell death containing ferroptosis, cuproptosis, autophagy, pyroptosis, etc (Chen et al., 2022; Liu et al., 2022). Additionally, it has been demonstrated that there exists a form of programmed and regulated cell death called necroptosis. Necroptosis is profit to defend pathogen invasion, and its inducements are mostly pathological changes or severe damage, presented as swelling and deformation of cell and organelles, rupture of membrane, random degradation of DNA. The dying cells releases damage-associated molecular patterns (DAMPs) and inflammatory cytokines stimulating the expression of proinflammatory genes in innate immune cells, and then drives inflammation (Frank and Vince, 2019; Lu et al., 2021; Mohammed et al., 2021; Newton et al., 2021) Necroptosis is distinct from classical apoptosis and necrosis and is regulated by membrane receptors and intracellular signal transduction molecules (Khoury et al., 2020; Yan et al., 2022). Several key molecules related to necroptosis, such as protein kinase 1 (RIPK1), receptor-interacting protein kinase 3 (RIPK3), and mixed-lineage kinase domain-like protein (MLKL), can be induced by various factors such as death receptors, interferons, toll-like receptors (TLR), intracellular DNA and RNA sensors, as well as other potential substances (Pasparakis and Vandenabeele, 2015; Grootjans et al., 2017; Weinlich et al., 2017). What is more important, research has shown that molecules related to necroptosis are differentially expressed in diseases and can serve as biomarkers (Li et al., 2022).

Hyperglycemia can also induce the development of DN by triggering necroptosis in renal tubular epithelial cells. Which is a significant mechanism underlying the pathogenesis of DN (Shen et al., 2022). Some researches state that necroptosis might play a critical role in the process of podocyte injury and reduction in DN (Erekat, 2022; Guo et al., 2023). Apoptosis of podocyte results in glomerular injury and podocyte depletion, causing proteinuria and Glomerular insufficiency. In patients with DN, the necroptosis is increased in tubulointerstitium and nephridial tissue of glomerulus, which is most obvious in the glomerulus in the stage of macroalbuminuria (Wang et al., 2022). In general, necroptosis plays an integral part of occurrence and development of DN. But there is currently no research on differential expression of necrosis related genes in DN and screening of biomarkers.

**FIGURE 1:**
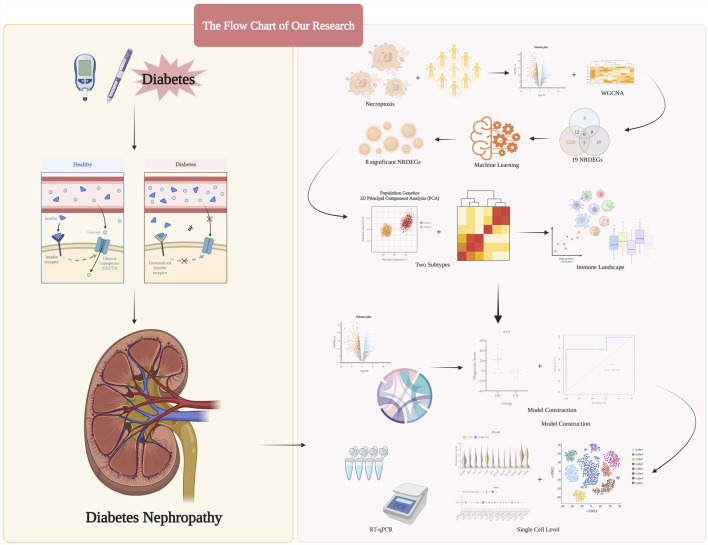
The step-by-step procedures and graph abstract of our study.

Herein, based on analysis of several public datasets obtained from the Gene Expression Omnibus (GEO), we selected key necroptosis-related regulators. Subsequently, combined with the above regulators, in our training set, all DN samples were categorized into two necroptosis-related subtypes with researching separately distinct immune infiltration landscapes and inflammatory statuses of them. Moreover, via the principal component analysis (PCA) algorithm, we calculated the necroptosis score to distinguish the above subtypes. Then, based on the above two subtypes, we exploited a necroptosis-mediated diagnostic model via various machine learning methods. Finally, the expression level of our eight significant regulators was validated in the single-cell level and several glomerulus samples. Overall, our research potentially provided methods in distinguishing different DN subtypes and indicated the novel insights in conducting personalized therapy strategies for DN patients, The step-by-step procedures of our study was exhibited in Figure 1.

## Materials and methods

### Data acquisition and processing

Based on the public and well-known large-scale database, GEO (https://www.ncbi.nlm.nih.gov/geo/), we searched and collected several data sets including patients with DN (Edgar et al., 2002). The inclusion criteria for data sets were as follows: 1) Organism: Homo sapines, 2) Tissue: Glomerulus, 3) datasets containing corresponding normal (CN) samples and comprehensive introduction for each sample. Moreover, the detailed introduction of the GEO data sets included in our research was exhibited in Table 1. In addition, the necroptosis-related regulators (NRGs) were downloaded from the public website GeneCards (https://www.genecards.org/), in which the selection criteria was the correlation score >1 and searched with the key word “necroptosis” (Safran et al., 2010).

**TABLE 1:** The information of the data sets utilized in our research.

Accession	Platform	Type	Samples		Tissue
			CN	DN	
GSE96804	GPL17586	Training Set	20	41	Glomerulus
GSE30528	GPL571	Validation Set	12	9	Glomerulus
GSE30122	GPL571	Validation Set	26	9	Glomerulus
			24	10	Tubules

### Differential expression analysis for our training set

To investigate the expression difference of genes in DN and explore the expression characteristic at the mRNA level, we utilized differential expression analysis via the “limma” package in R software, which was utilized to obtain the differentially expressed genes (DEGs) in our training set between CN and DN samples, respectively (Ritchie et al., 2015). The *p* < 0.05 were used to define the threshold of DEGs. To visualize the results of differential expression analysis, the “ggplot2” package in R software was used to create a volcano plot and a heat map.

### Weighted gene Co-expression network analysis (WGCNA)

The WGCNA was performed to screen the significant gene clusters correlated with DN (Langfelder et al., 2008). The “gplots” package in R software was utilized for hierarchical cluster analysis, and abnormal samples or values were deleted. The gene correlation between samples was calculated via WGCNA algorithm. Then we selected the best soft threshold power and established a standard non-proportional network. The WGCNA model or network was related to the characteristics of external samples. Via a dynamic tree-cutting strategy, various different modules named by different colors were constructed through the hierarchical clustering of genes.

### Machine learning (ML)

Several machine learning methods were used to investigate the diagnostic efficiency of the regulators. Random Forest (RF) can evaluate the significance of each feature in classification problems (Ishwaran and Kogalur, 2010; Wang and Zhou, 2017). Support vector machine recursive feature elimination (SVM-RFE), analgorithm that adds or removes features to obtain the optimal combination variable that maximizes model performance for specific feature variables. We use the “rfe” function in the R package “caret” for feature recursive elimination, set functions = caretFuncs, and obtain the best model. Moreover, least absolute shrinkage and selection operator-cox (Lasso-cox) regression analysis is an algorithm to evaluate the influence for binary outcome via merging some variables using the R package “glmnet” (Tang et al., 2017).

### Identification of different necroptosis-related subtypes of DN

According to the expression level of the selected significant necroptosis-regulators, we divided all the 41 DN samples in our training set into different necroptosis-related subtypes via the “ConsensusClusterPlus” package in R software (Wilkerson and Hayes, 2010). Then, with the most appropriate k-value, two different subtypes were identified.

Moreover, to exhibit the heterogeneity between the above subtypes, the PCA algorithm was performed (Diaz-Papkovich et al., 2021). And then, the necroptosis score was calculated with the results of PCA algorithm to explore the biological background of the two subtypes: **Necroptosis Score = Σ**_**i**_(**PCA1**_**i**_ **+ PCA2**_**i**_).

### Enrichment analysis

We utilized the ClueGO plug-in in the Cytoscape software with the threshold (*p* < 0.05) to conduct gene ontology (GO) enrichment analysis (Bindea et al., 2009). And the Molecular complex detection (MCODE) plug-in in cytoscape was used to extract several significant models in the gene-pathway interacted network of the results of ClueGO. Moreover, the R software package “clusterProfiler” was selected to perform the KEGG and Reactome enrichment analysis (Yu et al., 2012).

In addition, the gene set enrichment analysis (GSEA) was performed to comprehensively explore the biological background between the two necroptosis-related subtypes of DN. With the standard gene set of Hallmark, in the analysis, the size of the gene set is limited to 5–5,000 genes. Results with a *p*-value less than 0.05 were considered statistically significant. (Hacisalihoglu et al., 2016).

### Construction of a predictive diagnostic model based on the necroptosis-related subtypes of DN

Similarly and firstly, the differential expression analysis was performed between the two different necroptosis-related subtypes to obtain the DEGs (Hao et al., 2023). To select several more prominent genes to construct the diagnostic model, the criteria for identifying DEGs was the absolute value of log_2_ fold change (|log_2_ FC|) > 1 and *p* < 0.05.

Then, the Search Tool for the Retrieval of Interacting Genes (STRING, https://cn.string-db.org/) was used to construct the protein-protein interaction (PPI) network of our candidate genes and the cypscape software was utilized to visualized the PPI network (Shannon et al., 2003; Szklarczyk et al., 2023). Furthermore, we used the cyttoHubba plug-in in cytoscape software to screen the significant features from our candidate genes (Chin et al., 2014). Then, four machine learning methods: SVM-RFE, RF, univariate Cox regression analysis and Lasso-Cox regression analysis were conducted to further construct our diagnostic model. Finally, we performed multivariate logistics regression to establish our diagnostic model: **Diagnostic Score = Σ**_**i**_ (**Coefficient**_**i**_ * Expression level of feature_**i**_).

### Immune infiltration analysis

Single sample gene set enrichment analysis (ssGSEA) is a novel type of gene set variation analysis which is a method of unsupervised clustering based on a specific gene set to evaluate the score of each sample. In our study, ssGSEA was used to calculate the infiltration score of 28 different scores in DN. We collected genes from previous studies related to 28 different types of immune cells and predicted the infiltration abundance of immune cells based on these genes (Supplementary Table S1) (Barbie et al., 2009; Charoentong et al., 2017). Moreover, based on our expression matrix, we used the “IOBR” package in R software to select the ESTIMATE method to calculate the ESTIMATE Score of each sample in our training set (Yoshihara et al., 2013; Zeng et al., 2021). The CYT Score was calculated via averaging the expression value of GZMA and PRF1 in each sample to evaluate the inflammatory status. Furthermore, Mantel correlation analysis was performed to explore the correlated relationship between the significant necroptosis-related regulators and the infiltration score of each immune cell (Sunagawa et al., 2015).

### Single cell analysis

The Study: Human Diabetic Kidney: Wilson et al., PNAS 2019. (*Note: The CFH + cluster was renamed to parietal epithelial cells) in the public website: Humphreyslab (http://humphreyslab.com/SingleCell/) was included in our research to explore the expression trend of significant necroptosis-regulators in DN (Wilson et al., 2019).

### Animal experiments

Male 8-week-old diabetic db/db (BKS-Leprem2Cd479/Nju; n = 8) and their nondiabetic wild type (C57BL/Ksj; n = 8) mice were purchased from the Model Animal Research Center of Nanjing University (Nanjing, China). All mice were housed under specific pathogen-free conditions with controlled temperature and humidity (22°C ± 2°C, 50% ± 5% RH) and a standard 12-h light/dark cycle. All experimental procedures were approved by the Institutional Animal Care and Use Committee at Nanchang University (NO. 0064257) and performed in accordance with the guidelines for the ethical treatment of laboratory animals. All mice were randomly divided into two groups: control (CN, n = 8) group and diabetic nephropathy (DN, n = 8) group. The successful establishment of the diabetic model was defined as random blood glucose levels ≥16.7 mmol/L. At 20 weeks of age, the mice were anesthetized with isoflurane and subsequently euthanized. *Postmortem*, the body weight, kidney weight, and tibial length were measured. The kidneys were then excised and divided into sections for further analysis.

### Reverse transcription-quantitative PCR (RT-qPCR)

The total RNA of serum was extracted from fresh glomerulus samples utilizing Trizol reagent (Ambion, Singapore). The quantity and purity of the total RNA were determined using the Nanodrop®ND1000 (TIANJIN). The total RNA was reverse transcribed into complementary DNA (cDNA) employing the Script cDNA synthesis kit (TianGEN). Quantitative PCR reaction was performed on an EDC-810 Real-Time PCR Detection Instrument (Eastwin life, China), using SYBR Premix ExTaq kit (Takara, China), All genes were analyzed Relatively and calibrated to the expression of control groups. The 2^^−ΔΔCt^ method was employed to calculate the relative RNA expression values. The primer pairs used for the amplification of target genes are listed in Supplementary Table S2. All the above experiments were strictly carried out in accordance with the instructions.

### Biochemical assays

Serum blood urea nitrogen (BUN) levels were quantified in murine samples using a commercial colorimetric assay kit (DEIABL-M3) per the manufacturer’s protocol. An enzymatic creatinine reagent kit (DEIABL-M4S) was employed to determine serum creatinine concentrations in the sourced murine serum samples. Urine specimens harvested from the mice underwent analysis of microalbumin content by enzyme-linked immunosorbent assay utilizing a microalbumin-specific antibody (ab108792). Murine urine creatinine concentrations were evaluated by a commercial colorimetric assay kit (LS-F13025) following the provided manufacturer guidelines.

### Statistical analysis

The data were shown as mean ± SD. R software (version 4.2.2) and its related software packages were used to process and analyze data. The unpaired *t*-test was utilized to analysis the statistical difference between two different groups. *p* < 0.05 were considered statistically significant. And the “RMS” package in R software was used to visualize Nomograms. The receiver operating characteristic (ROC) curves were visualized via the Sangerbox website (http://vip.sangerbox.com/home.html).

## Results

### General exploration of NRGs in DN

**FIGURE 2:**
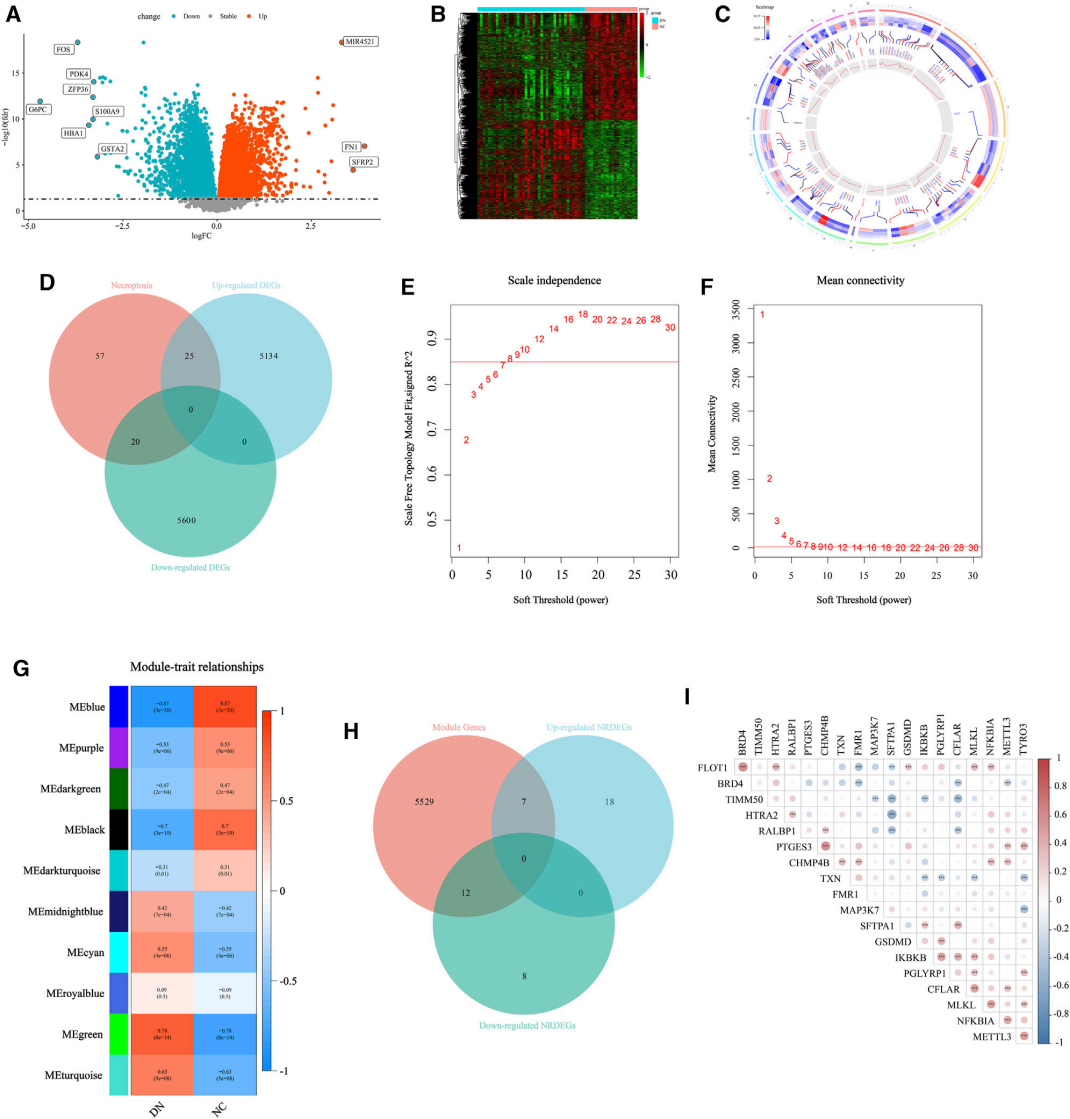
Selection for the NRDEGs between DN and CN. **(A)** The volcano map for the DEGs identified between DN and CN with *p < 0.05*. **(B)** The heat map exhibited the detailed expression status of the above DEGs. **(C)** The location of the upregulated DEGs with the top highest log_2_FC and the downregulated DEGs with the lowest log_2_FC in the chromosomes with the corresponding p and normalized expression value. **(D)** The Venn-gram was utilized to describe the relationship between NRGs, upregulated DEGs and downregulated DEGs. **(E,F)** The determination for the soft threshold of WGCNA. **(G)** The gene modules calculated via WGCNA with the corresponding p and correlation coefficient. **(H)** The Venn-gram was utilized to intersect the module genes, upregulated NRDEGs and downregulated NRDEGs. **(I)** The correlated heat map of the 19 NRDEGs in DN samples of our training set. *, *p < 0.05*; **, *p < 0.01*; ***, *p < 0.001*.

Firstly, to exhibit the remarkable characteristic of necroptosis in the transcriptomic level in DN. Initially, we conducted a differential expression analysis comparing the NC group and DN group. Genes with a *p*-value less than 0.05 were identified as differentially expressed genes (DEGs), as depicted in Figure 2A. A total of 10,779 DEGs were identified, with 5,159 genes being upregulated and 5,620 genes being downregulated. The detailed expression status of these DEGs is displayed in Figure 2B, providing a comprehensive overview of their differential expression patterns. Then, the location in chromosomes of the top 50 downregulated and upregulated genes were listed in Figure 2C with the corresponding *p-value* and normalized expression value. The primary NRGs in DN were obtained via intersecting the upregulated DEGs, downregulated DEGs, and NRGs in Genecards. Finally, a total of 45 necroptosis-related DEGs (NRDEGs) were identified (Figure 2D).

Furthermore, the WGCNA was performed to extract several gene modules significantly correlated with DN (Figures 2E–G). We intersected the above NRDEGs and the genes in the significant modules of WGCNA to ensure several NRDEGs prominently correlated with DN (Figure 2H). As a result, a total of 19 NRDEGs were identified. Moreover, a Spearman correlation analysis was performed among the above 19 NRDEGs and almost all regulators indicated a remarkably positive correlation (Figure 2I).

### Further screening of the significant NRDEGs in DN via ML

**FIGURE 3:**
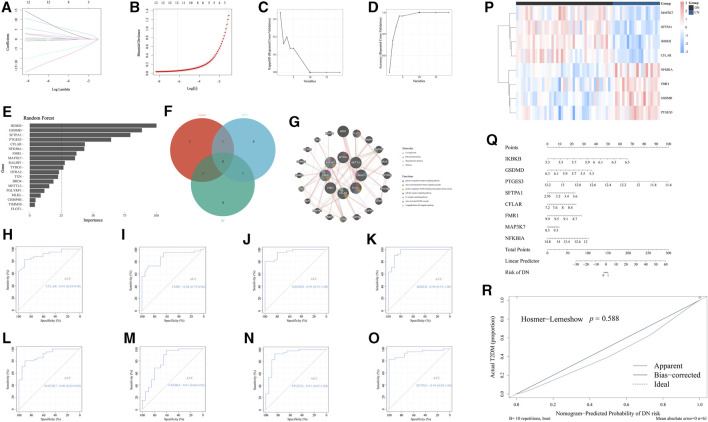
Identification of the significant NRDEGs in DN. **(A,B)** The outcome of the Lasso-cox regression analysis. **(C,D)** The results of the SVM-RFM. **(E)** The importance of the 19 significant NRDEGs calculated via the random forest algorithm. **(F)** The Venn-gram exhibited the intersection of the results of the above 3 ML methods. **(G)** The co-expression network of the eight significant NRDEGs. **(H-O)** The ROC curves of **(H)** CFLAR, **(I)** FMR1, **(J)** GSDMD, **(K)** IKBKB, **(L)** MAP3K7, **(M)** NFKBIA, **(N)** PTGES3, and **(O)** SFPTA1 in the diagnosis of DN. **(P)** The heat map revealed the detailed expression status of the eight significant NRDEGs in all samples of our training set. **(Q)** The nomogram of the eight significant NRDEGs in predicting the risk of DN. **(R)** The calibration curve of the above nomogram.

According to the above general exploration of the role of necroptosis-related regulators in DN, we subsequently excavated several significant NRDEGs in DN to evaluate the crucial characteristic of necroptosis in DN. To begin with, we performed the Lasso regression analysis to obtain the key features in distinguishing DN and NC (Figures 3A, B). Moreover, another ML method, SVM-RFE was performed to extract the features with prominent diagnostic efficiency. As a result, we obtained 10 NRDEGs (Figures 3C, D). Via the cross validation of a 10×fold, a total of 12 NRDEGs were identified. Furthermore, RF was utilized to assess the importance of 19 NRDEGs in recognizing NC and DN patients. With the importance >25 as the criteria, 10 NRDEGs were selected (Figure 3E). Finally, after intersecting results of the above 3 different ML methods, a total of 8 NRDEGs (CFLAR, FMR1, GSDMD, IKBKB, MAP3K7, NFKBIA, PTGES3, and SFTPA1) were finally regarded as the significant NRDEGs (Figure 3F). The co-expression network of the eight significant NRDEGs was construction with co-expression of 61.91%, physical interactions of 28.79%, shared protein domains of 7.12%, and pathway of 2.17%, wherein the enrichment analysis revealed that the 8 regulators was mainly associated with the inflammatory and immunal pathways, such as “pattern recognition receptor signaling pathways” “toll-like receptor signaling pathways”, and “I-kappaB kinase/NF-kappaB signaling” (Figure 3G).

Moreover, to better demonstrate the diagnostic characteristic of the above 8 regulators, the ROC curves were exhibited, the area under curve (AUC) of which were 0.96 0f IKBKB, 0.95 of GSDMD, 0.91 of PTGES3, 0.94 of SFTRA1, 0.91 of CFLAR, 0.88 of FMR1, 0.90 of MAP3K7 and 0.81 of NFKBIA, which emphasized the powerful diagnostic efficiency (Figures 3H–O). Meanwhile, the heat map exhibited the expression level of the above 8 features between CN and DN (Figure 3P). Furthermore, utilizing the expression levels of the eight features in GSE96804, we developed a nomogram to visualize the predictive ability of our model for diagnosing DN patients (Figure 3Q). Additionally, the calibration curve was generated to assess the accuracy and calibration of our model (Figure 3R).

### Obtaining of two different necroptosis-related subtypes of DN

**FIGURE 4:**
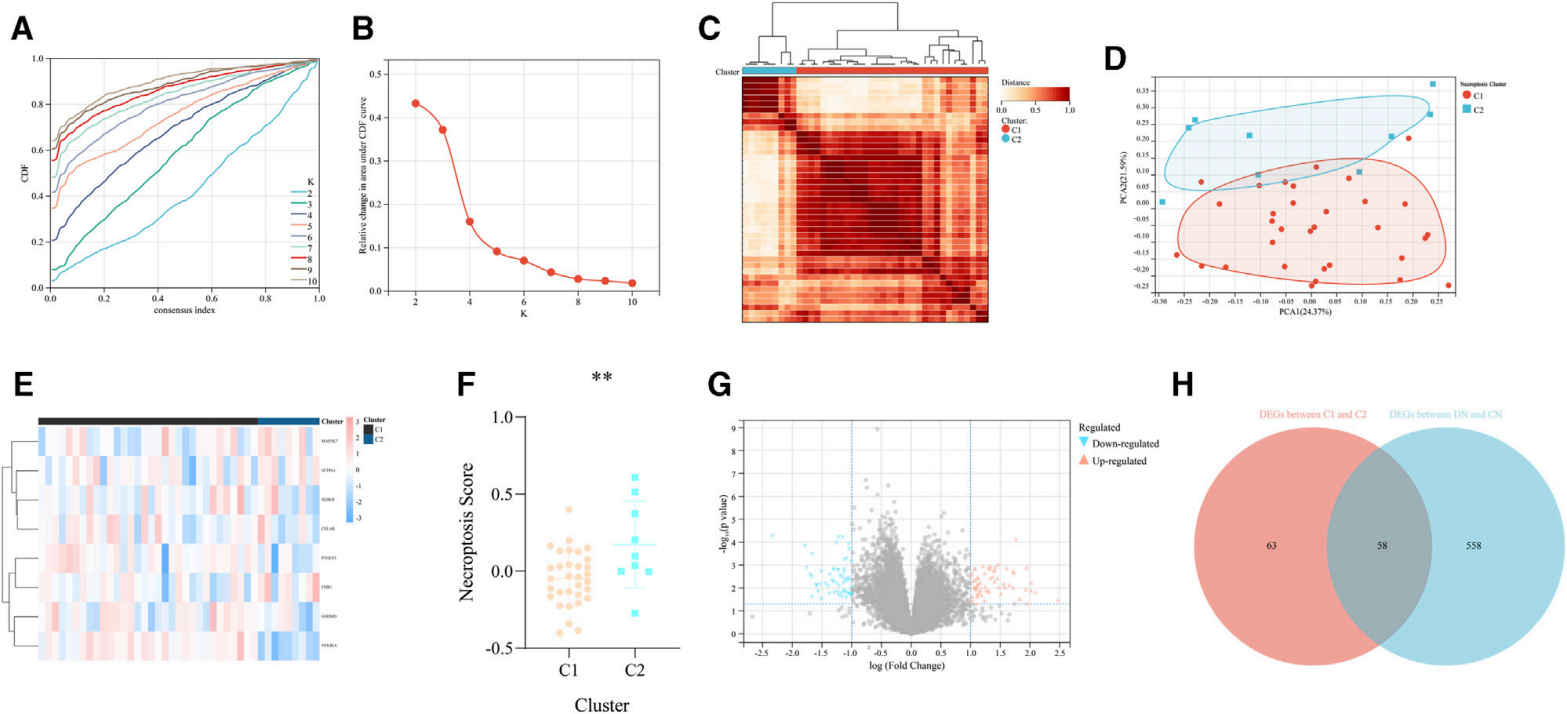
The recognition of two different necroptosis-related subtypes of DN. **(A)** The cumulative distribution curve of each k value from 2 to 10 in the consensus cluster analysis. **(B)** The area under the curve of each k value. **(C)** The heat map of the two different necroptosis-related subtypes of DN. **(D)** The PCA diagram exhibited the general distribution of the above two subtypes. **(E)** The heap map of the eight significant NRDEGs in the two subtypes. **(F)** The difference of the necroptosis score calculated via the PCA diagram between the above two subtypes. **(G)** The volcano map was utilized to identify the DEGs between the above two subtypes. **(H)** The Venn-gram revealed the intersection of the DEGs between the above two subtypes and the DEGs between DN and CN.

According to the expression value of the above 8 significant NRDEGs (IKBKB, GSDMD, PTGES3, SFTRA1, CFLAR, FMR1, MAP3K7, and NFKBIA) in DN samples, all the 41 DN samples in our training set were classified into two various necroptosis-related subtypes (C1 and C2) with the most compatible K-value (K = 2) using a consensus cluster analysis (Figures 4A–C). Moreover, the PCA-gram exhibited an obvious differentiation between the above two DN subtypes (Figure 4D). In addition, the detailed expression level of all our 8 crucial NRDEGs between the two DN subtypes were exhibited in the heat map, which emphasized the heterogeneity of our two subtypes (Figure 4E). Meanwhile, via the PCA algorithm, we calculated the necroptosis-score to objectively recognize the two necroptosis-related subtypes, in which subtype C1 was prominently lower than subtype C2 (*p* < 0.05, Figure 4F).

### Exploration of the biological differential characteristic between two necroptosis-related subtypes

According to the above analysis, the significant difference between the two necroptosis-related subtypes has been exhibited. Thus, we further investigated the biological differential characteristic between the two subtypes. First, we performed differential expression analysis to obtain the DEGs between the two subtypes (C2 vs. C1). As a result, 121 DEGs were identified based on the criteria of *p* < 0.05 and |log_2_FC| > 1 (Figure 4G). Similarly, differential expression analysis was performed in the whole training set to determine DEGs between CN and DN group, which results in a total of 616 DEGs, including 290 upregulated and 326 downregulated. Moreover, to select some candidate features with potential diagnostic efficiency, we intersected the DEGs between two necroptosis-related subtypes and the DEGs between CN and DN group (Figure 4H).

Subsequently, to better exhibit the biological characteristic between the two subtypes, we performed GO and KEGG enrichment analysis for the 58 candidate features. Via ClueGO plug-in in Cytoscape software, the GO enrichment analysis revealed that pathways related to metabolism and immunity played a crucial role in necroptosis-related subtypes, especially “hemoglobin complex” with the proportion of 38.46% and “complement activation, alternative pathway” with the proportion of 23.08% (Supplementary Figure S1A). Moreover, the MCODE plug-in was utilized for the extract of some significant functional clusters and these clusters were mainly related to metabolism and immunity (Supplementary Figures S1B–D). Meanwhile, the results of KEGG and Reactome enrichment analysis proved the crucial role of immune landscape in the two necroptosis-related subtypes (Supplementary Figures S1E, F). Moreover, the GSEA enrichment analysis was also performed to further explore the functional backgrounds between the two subtypes, the results of which emphasized the role of “DNA Repair”, “TGF-β Signaling”, and “Heme Metabolism” (Supplementary Figures S1G–I).

### The construction of a diagnostic model based on the two necroptosis-related clusters

**FIGURE 5:**
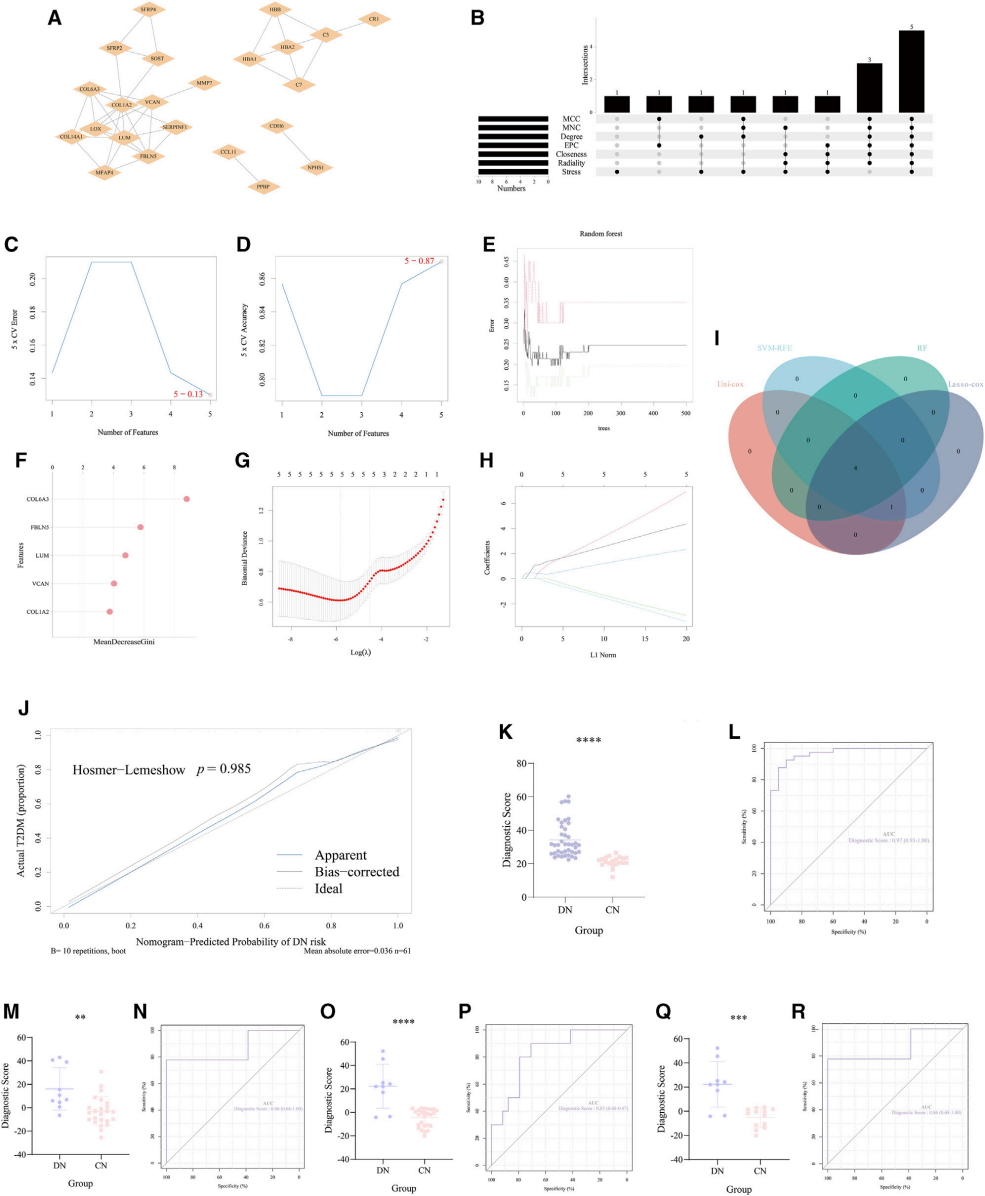
Construction and validation of the necroptosis-mediated diagnostic model for DN. **(A)** The PPI network of the 35 overlapped DEGs. **(B)** The upset diagram revealed the top 15 hub DEGs in the seven algorithms of cyttoHubba plug-in. **(C,D)** The results of the SVM-RFE. **(E,F)** The results of the random forest. **(G,H)** The outcome of the Lasso-cox regression analysis. **(I)** The Venn-gram exhibited the intersection of the 4 ML methods in identifying the elements for constructing our diagnostic model. **(J)** The calibration curve of the diagnostic score calculated via our model. **(K, M, O, Q)** The difference of the diagnostic score in **(K)** GSE96804, **(M)** the glomerulus tissue in GSE30122, **(O)** the tubules tissue in GSE 30122 and **(Q)** GSE30528. **(L, N, P, R)** The ROC curve of the diagnostic score in **(L)** GSE96804, **(N)** the glomerulus tissue in GSE30122, **(P)** the tubules tissue in GSE 30122 and **(R)** GSE30528. **, *p* < 0.01; ***, *p* < 0.001; ****, *p* < 0.0001.

According to the crucial role of significant NRDEGs in DN and the lack of effective diagnostic markers or models, we exploited a novel necroptosis-mediated diagnostic model for DN patients. Via the above rounds of selection, we finally ensured 58 genes for the construction of our model. Firstly, all the 58 features were imported into the STRING website to establish a PPI network (Figure 5A). Then via the cyttoHubba plug-in in Cytoscape software, we utilized 7 algorithms to obtain the top 15 features with highest interaction score in each algorithm (MCC, MNC, Degree, EPC, Closeness, Radiality, Stress). After intersecting the 10 features in each algorithm, a total of 5 features were identified in all the 7 algorithms, which were selected for the subsequent analysis (Figure 5B).

Furthermore, we utilized 4 different ML methods to select several significant features. Originally, one classical ML methods, SVM-RFE, was performed to screen features with more accurate diagnostic efficiency. As a result, all the 5 features were regarded as regulators with significant diagnostic efficiency (Figures 5C, D). Then, we utilized an additional ML method (RF) to calculate the importance of all the 5 features in recognizing the DN and CN samples. The lollipop chart revealed the importance in detail and 4 features with importance >4 were regarded significant features (Figures 5E, F). Moreover, to ensure the accuracy of our selection, we further performed another ML method, lasso-cox regression analysis to extract the features, which can better distinguish DN and CN patients. Five features were considered to play a key role in the diagnosis of DN (Figures 5G, H). In addition, the univariate-cox regression analysis was also conducted to select the significant features with the criteria: *p* < 0.05 (Table 2). Finally, the 4 features (COL6A3, FBLN5, LUM, and VCAN) intersected in the whole 4 ML methods were regarded as the source elements of the construction of our diagnostic model (Figure 5I).

**TABLE 2:** The results of the univariate-cox regression analysis.

Features	Hazard ratio	95%CI lower	95%CI higher	*p*-value
COL1A2	97.11	4.29	2,198.66	0.004
COL6A3	542.4	10.05	29,262.06	0.002
FBLN5	2.75	1.51	5.04	0.001
LUM	2.86	1.6	5.13	0.0004
VCAN	2.03	1.25	3.29	0.0044

Subsequently, for establishing our diagnostic model for DN, the logistic regression analysis was performed: **Diagnostic Score = (12.1605262153362 × COL6A3) + (-3.03974790703008 × FBLN5) + (2.42256407679845 × LUM) + (-3.13831314409003 × VCAN)**. And the calibration curve revealed that there was no potential difference between our model and the ideal situation (*p = 0.985*, Figure 5J). In our training set (GSE96804) we performed unpaired *t*-test to inspect the statistical significance between CN and DN group based on the formula of the diagnostic score described above. As a result, our necroptosis-mediated diagnostic model revealed a statistically significant difference in the training set (*p < 0.0001*, Figure 5K). Meanwhile, the AUC of our model in the training set was 0.97, which emphasized the powerful diagnostic efficiency (Figure 5L). Additionally, to strengthen the reliability of our diagnostic model, we chose two external verification sets (GSE30528 and GSE30122) to validate our model. Interestingly, in GSE30528 and GSE30122, the statistical significance between CN and DN group both occurred (Figures 5M, O, Q). And the AUC was 0.86 the glomerulus tissue in GSE30122, 0.83 the tubules tissue in GSE 30122 and 0.86 in GSE30528, which indicated the accuracy of the diagnostic efficiency of our model (Figures 5N, P, R).

### The investigation of the immune landscape in the two necroptosis-related clusters

**FIGURE 6:**
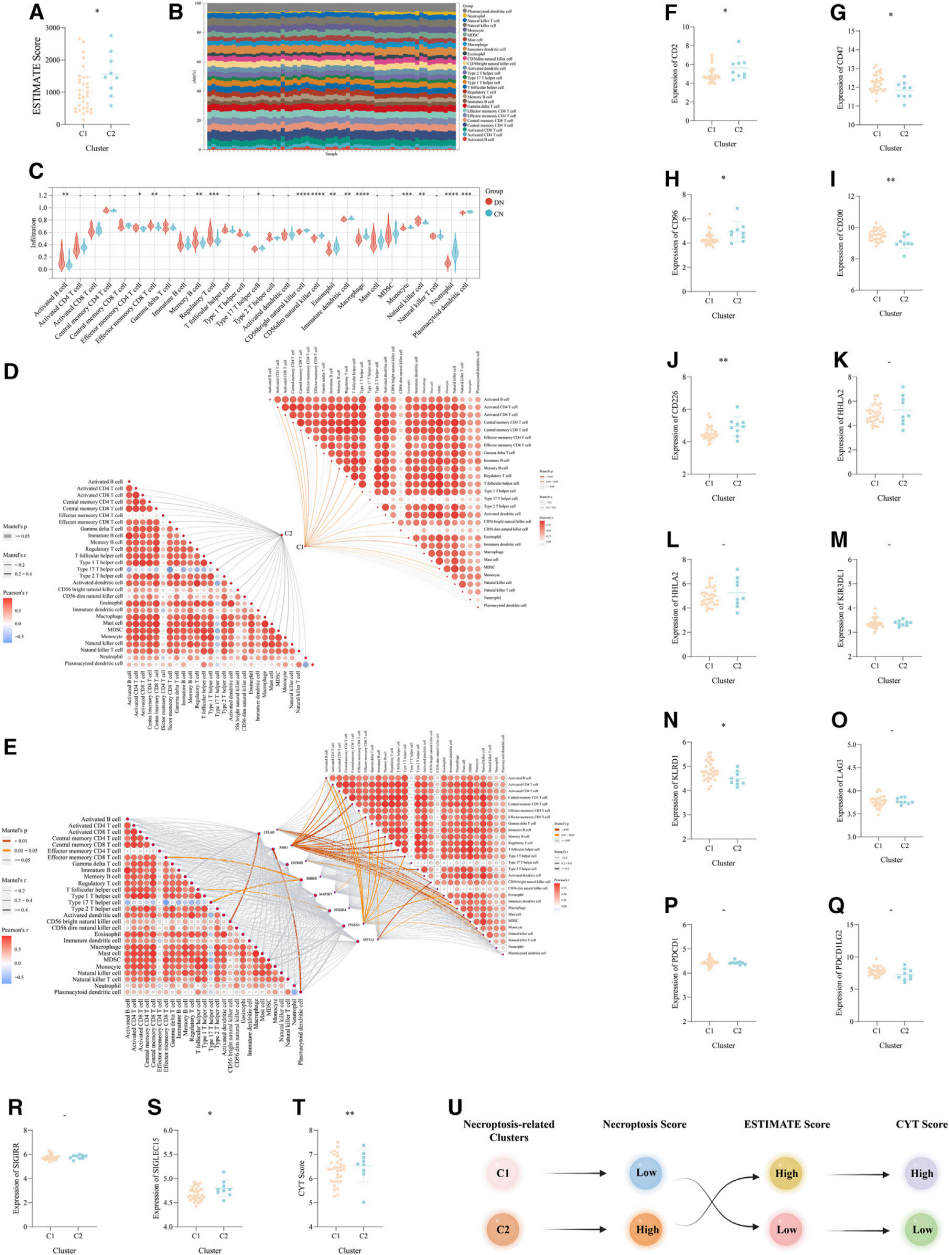
The immune landscape and microenvironment regulated by necroptosis in DN. **(A)** The difference of the ESTIMATE score between the above two necroptosis-related subtypes. **(B)** The detailed proportion of each immune cells in each sample of our training set. **(C)** The difference of the infiltration score of each immune cells calculated via ssGSEA between DN and CN. **(D,E)** The mantel correlation heat map between **(D)** the necroptosis score and the infiltration score of the 28 kinds of immune cells, **(E)** the expression level of eight significant NRDEGs and the infiltration score of the 28 kinds of immune cells. **(F-S)** The expression difference of **(F)** CD2, **(G)** CD47, **(H)** CD96, **(I)** CD200, **(J)** CD226, **(K)** CTLA4, **(L)** HHLA2, **(M)** KIL3DR1, **(N)** KLRD1, **(O)** LAG3, **(P)** PDCD1, **(Q)** PDCD1LG2, **(R)** SIGIRR, **(S)** SIGLEC15 between the above two subtypes. **(T)** The difference of CYT score between the above two subtypes. **(U)** The general relationship among the necroptosis-related subtypes, immune landscape and inflammatory response. -, *p > 0.05*; *, *p < 0.05*; **, *p < 0.01*; ***, *p < 0.001*; ****, *p < 0.0001.*

Based on the above functional exploration of the two necroptosis-related clusters, the immune microencironment was considered to keep a critical role in DN and corresponding clusters. Two different algorithms, ESTIMATE and ssGSEA, were utilized to comprehensively explore the immune microenvironment of DN. Via the ESTIMATE algorithm, the ESTIMATE Score was lower in C1 than that in C2, which revealed there were potentially different immune response and infiltration between the above two necroptosis-related subtypes of DN (Figure 6A). Then, via ssGSEA, we visualized the superficial landscape of various immune cells infiltrations in all samples of our training set, which exhibited the prominent difference of immune microenvironment between CN and two different DN clusters (Figure 6B). And between DN and CN, abundant immune cells exhibited significant infiltration status (Figure 6C). Moreover and intriguingly, we concuded the Mantel correlation analysis to compute the correlation coefficient between the expression level of our 8 significant NRDEGs and the immune infiltration score. As a result, the necroptosis score and expression of 8 NRDEGs in C1 was more correlated with the immune microenvironment than in C2, further proving the result of ESTIMATE (Figures 6D, E).

According to the above investigation of the immune landscape in two different clusters, we ensure the diversified immune microenvironment between C1 and C2. And based on the significant role of immune checkpoints (ICKs) in the regulation of immune response, we further explore the role of ICKs in DN. The unpaired *t*-test was used to detect the expression of 14 classical ICKs between C1 and C2. Interestingly, a total of 8 ICKs exhibited a significantly different expression in C2 than C1 (). Moreover, the CYT Score calculated via the average of the expression of GZMA and PRF1 also exhibited the different inflammatory status between the two necroptosis-related subtypes of DN (Figure 6T). Finally, the Sankey plot demonstrated the potential relationship between the two necroptosis-related subtypes, the immune microenvironment and the inflammatory status (Figure 6U).

### Exploration of the expression of the eight significant NRDEGs in the single-cell Level

**FIGURE 7:**
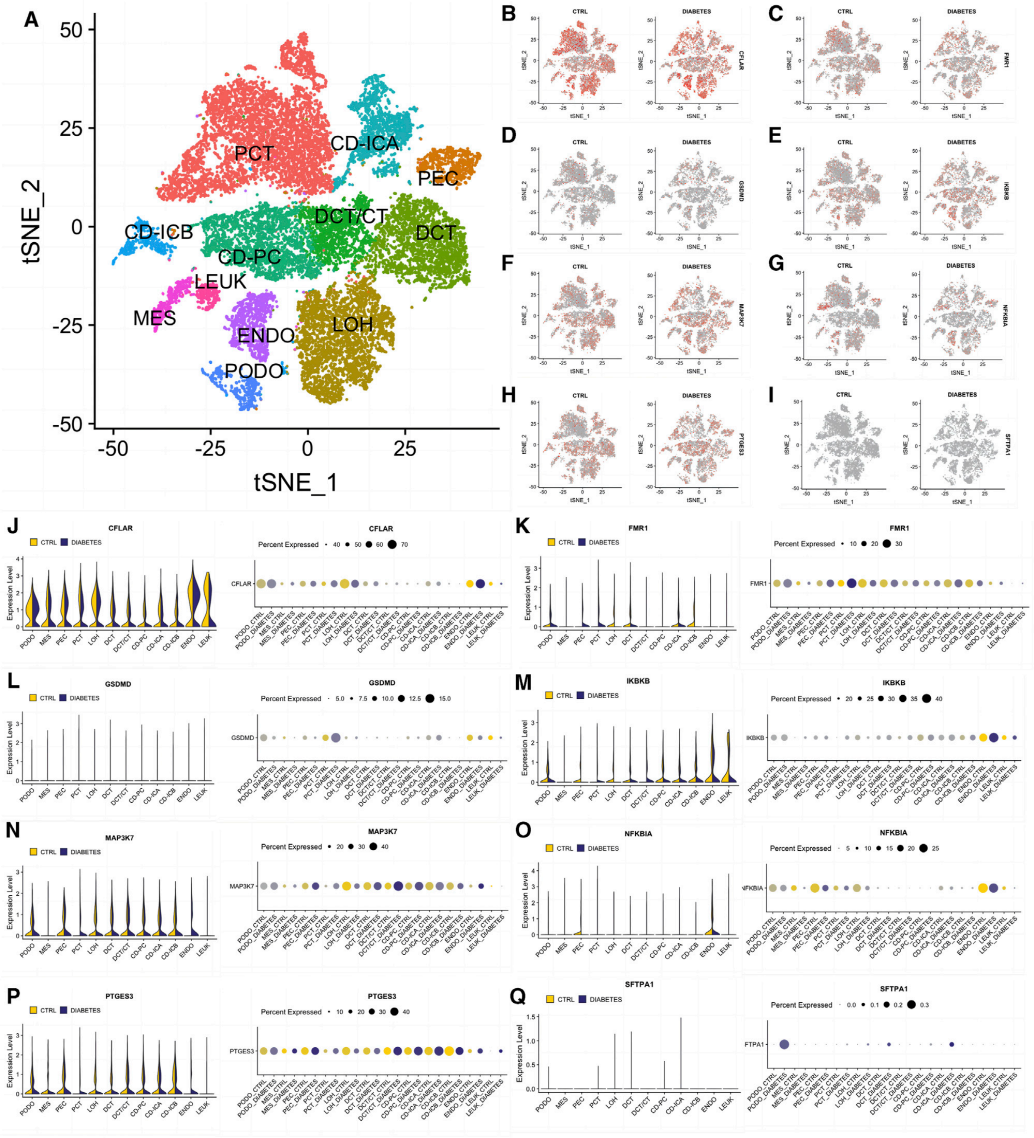
The expression status of our eight significant NRDEGs in the single-cell dataset. **(A)** The annotated heat map of different cell clusters. **(B–I)** The expression cluster heat map of **(B)** CFLAR, **(C)** FMR1, **(D)** GSDMD, **(E)** IKBKB, **(F)** MAP3K7, **(G)** NFKBIA, **(H)** PTGES3 and **(I)** SFTPA1 between CN and DN. **(J-Q)** The detailed expression of **(J)** CFLAR, **(K)** FMR1, **(L)** GSDMD, **(M)** IKBKB, **(N)** MAP3K7, **(O)** NFKBIA, **(P)** PTGES3 and **(Q)** SFTPA1 in each cell between CN and DN exhibited via violin plot and bubble plot. PCT, proximal convoluted tubule; CFH, complement factor H; LOH, loop of Henle; DCT, distal convoluted tubule; CT, connecting tubule; CD, collecting duct; PC, principal cell; IC, intercalated cell; PODO, podocyte; ENDO, endothelium; MES, mesangial cell; LEUK, leukocyte.

To better describe the expression pattern of our 8 significant NRDEGs in DN, we selected a data set including 12 different cell clusters (PCT, CD-ICA, PEC, DCT, DCT/CT, CD-PC, CD-ICB, MES, LEUK, ENDO, LOH, PODO, n = 23,980). The general landscape of the above 12 cell clusters in different samples was exhibited in Figure 7A. Interestingly, the expression level of our 8 significant NRDEGs (CFLAR, FMR1, GSDMD, IKBKB, MAP3K7, NFKBIA, PTGES3, and SFTPA1) was different in the different cells between the DN and CN group, which revealed that our 8 significant NRDEGs played a crucial and various role in the characteristic of DN (Figures 7B–Q).

#### Validation of the eight significant NRDEGs and exploration of the correlation with the kidney function

**FIGURE 8:**
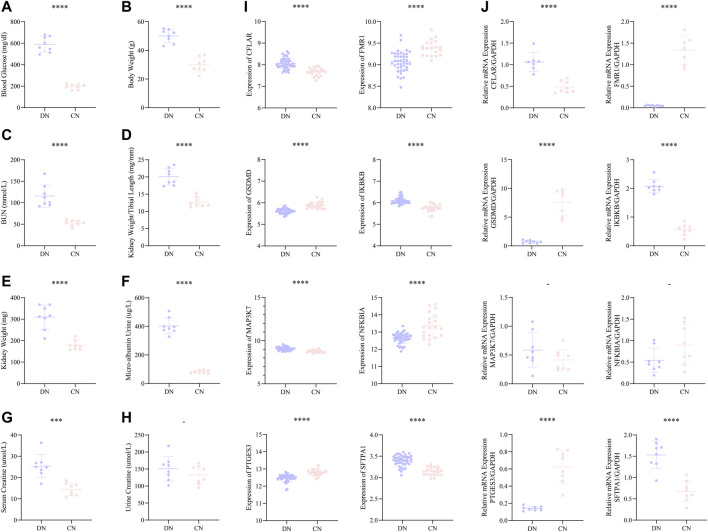
**(A–H)** The statistical difference of **(A)** blood glucose, **(B)** body weight, **(C)** BUN, **(D)** kidney weight/tibial length, **(E)** kidney weight, **(F)** micro-albumin urine, **(G)** serum creatine, and **(H)** urine creatine between the DN and CN group, indicating the successful construction of our DN mice model. **(I)** The expression difference of the above eight significant NRDEGs between DN and CN in the training set. **(J)** The expression difference of the above eight significant NRDEGs between DN and CN in the DN mice model via RT-qPCR. -, p > 0.05; ****, p < 0.0001.

Moreover, we constructed a mice model with DN to better and comprehensively explore and validate the above-mentioned eight significant NRDEGs. In our mice model, all the eight indexes, including blood glucose, body weight, blood urea nitrogen (BUN), kidney length/tibial length, kidney weight, micro-albumin urine, serum creatine, and urine creatine, exhibited a significantly higher trend in the DN group except the urine creatine (Figures 8A–H). And the volume of the kidney in the DN group was also lager than that in the CN group (Supplementary Figure S2). The above phenomenon indicated the success of our DN model. We firstly extracted the expression level of the eight significant NRDEGs between the DN and CN group in our training set (Figure 8I). And the glomerulus samples were collected form the mouse model with DN or healthy mouse model. After obtaining the glomerulus tissue from each mouse, the RT-qPCR was performed to detect the concentration of our eight significant NRDEGs. As a result, except MAP3K7 and NFKBIA, the other six NRDEGs exhibited significant expression difference between DN and CN group and consistent expression trend with the phenomena in our bioinformatic analysis (Figure 8J).

Furthermore, we explored the role of the eight significant NRDEGs in the kidney role. The spearman correlation analysis between the expression level of the eight significant NRDEGs and the eight indexes in our DN model. In Table 3, interestingly, the low-expressed genes in DN (FMR1, GSDMD, NFKBIA, and PTGES3) were significantly negatively correlated with almost all the indexes while the high-expressed genes in DN (CFLAR, IKBKB, MAP3K7, and SFTPA1) were positively correlated with the indexes.

**TABLE 3:** The correlation between the expression level of the 8 significant NRDEGs and the several indexes related to our DN model.

Variable	Micro-albumin urine		Urine creatine		Serum creatine		BUN		Blood glucose		Kidney weight/Tibial length		Kidney weight		Body weight	
	Coe	*p*-value	Coe	*p*-value	Coe	*p*-value	Coe	*p*-value	Coe	*p*-value	Coe	*p*-value	Coe	*p*-value	Coe	*p*-value
NFKBIA	−0.396	0.144	−0.271	0.328	−0.118	0.676	−0.402	0.137	−0.331	0.229	−0.627	0.012	−0.312	0.258	−0.381	0.162
FMR1	−0.929	<0.0001	−0.157	0.577	−0.74	0.002	−0.828	<0.0001	−0.919	<0.0001	−0.822	<0.0001	−0.835	<0.0001	−0.901	<0.0001
PTGES3	−0.861	<0.0001	−0.254	0.361	−0.643	0.01	−0.776	0.001	−0.864	<0.0001	−0.757	0.001	−0.817	<0.0001	−0.79	<0.0001
GSDMD	−0.771	0.001	−0.095	0.737	−0.529	0.042	−0.681	0.005	−0.733	0.002	−0.767	0.001	−0.566	0.028	−0.694	0.004
MAP3K7	0.22	0.43	−0.14	0.62	0.488	0.065	0.256	0.357	0.417	0.122	0.213	0.447	0.482	0.069	0.425	0.114
CFLAR	0.634	0.011	0.145	0.607	0.477	0.073	0.304	0.271	0.517	0.048	0.666	0.007	0.559	0.03	0.529	0.043
SFTPA1	0.813	<0.0001	0.409	0.13	0.497	0.059	0.691	0.004	0.739	0.002	0.802	<0.0001	0.669	0.006	0.783	0.001
IKBKB	0.929	<0.0001	0.176	0.531	0.795	<0.0001	0.812	<0.0001	0.938	<0.0001	0.854	<0.0001	0.859	<0.0001	0.922	<0.0001

## Discussion

DN is a clinical syndrome, which is characterized by albuminuria, or increased excretion of urine albumin (Doshi and Friedman, 2017). As one of the most common causes of end-stage renal disease, the hazard of DN to homeostasis and endocrine system is irreparable. However, over the last abundant years, most studies have considered DN as an endocrine and microvascular disease (Flyvbjerg, 2017; Li X. et al., 2022). Actually, DN has been regarded as a chronic inflammatory disease in recent years, in which immune system and inflammatory cytokines played a critical role (Yu et al., 2021; Chen et al., 2022). In addition, with the continuous deepening of research on various diseases at the cellular level and molecular mechanisms, more and more studies indicated programmed cell death as the potential culprit of various diseases (Kopeina and Zhivotovsky, 2022). Necroptosis, an emerging form of programmed cell death differed from necrosis and apoptosis, induces the occurrence of inflammation via attacking the cytoplasmic membrane. Cells with necroptosis expel their contents, stimulating inflammatory responses in surrounding cells and activating the body’s immune response (Abi-Aad et al., 2019; Bertheloot et al., 2021). Moreover, evidence indicate that necroptosis may play a crucial role in the damage and decline of cells in DN, especially since it has been shown to be triggered after hyperglycemia-induced inhibition of apoptosis (Liu et al., 2018). In the above exploration and analyses, we have superficially described the correlation between necroptosis and DN. Despite a growing interest in this field, major research gaps remain that need to be addressed. Herein, we tried to create a new prospect for the tip of an iceberg in the field of the diagnosis and personalized therapy for DN.

In our study, a RNA-seq dataset, GSE96804, including 41 DN samples and 20 CN samples, was obtained from the GEO as our training set. A total of 102 necroptosis-related genes were included, in which 45 were regarded as the necroptosis-related DEGs. Moreover, for DN, as a metabolic disease, various pathways play a crucial role in the development and occurrence of DN. Herein, the percentage of necroptosis-related DEGs in the necroptosis-related genes also demonstrated the significance in DN. Via differential expression analysis and WGCNA, a total of 19 NRDEGs were identified. Then, various ML methods were performed to extract the final 8 significant NRDEGs (CFLAR, FMR1, GSDMD, IKBKB, MAP3K7, NFKBIA, PTGES3 and SFTPA1). Subsequently, the diagnostic efficiency of the above 8 features were exhibited. Moreover, based on the expression pattern of the 8 features, we classified 41 DN samples into two distinct necroptosis-associated subtypes. The PCA algorithm was utilized to clarify the difference between the two subtypes and calculate the necroptosis score to excavate and correlated the biological background of the subtypes. In addition, via the discussion of the correlation between the immune/inflammatory response and the necroptosis score, we inferred the subtype with higher necroptosis score may be faced with more severe immune infiltration and inflammatory response. Additionally, to better emphasize the characteristic of our necroptosis-associated subtypes, we constructed a necroptosis-mediated diagnostic model, which was validated in several external datasets. Finally, all the above 8 significant NRDEGs were validated in the single-cell dataset and the glomerulus tissue of the mouse model with DN via RT-qPCR.

Necroptosis is a pro-inflammatory programmed cell death via attacking the cytoplasmic membrane, which is considered as a prominent inducing factor of inflammation (Bertheloot et al., 2021). Zhu et al. prestented that RIPK3-related necroptosis played a crucial role in the renal tubular epithelial cell death of chronic renal injury (Zhu et al., 2020). Interestingly, it has been confirmed that hyperglycemia in diabetes patients can activate the intrarenal RAAS system, which induced the release of the renin and angiotensin (Ang) (Sharma et al., 2006; Lovshin et al., 2018). However, high concentration of AngⅡ may result in potential cytotoxicity to induce the renal tubular cell necrosis via activating the Fas and FasL (Basta et al., 2004). Meanwhile, the depletion of podocytes is another main cause of DN (Erekat, 2022). The research of Xu et al. discovered that the abnormal high expression of UCHL1 in the podocytes of DN disturbed the ubiquitination of the RIPK1/RIPK3 pathway, which finally induced the occurrence of necroptosis (Xu et al., 2019). Moreover, in our research, the pathways related to “hemoglobin complex” and “complement system” was regarded as the two significant biological process in the two necroptosis-related subtypes of DN. Recent studies demonstrated that there have been indivisible relationships between necroptosis and complement. Necroptosis was confirmed that induces the injury of vessels via complement activation and alternative complement pathways (Schreiber et al., 2017). Meanwhile, the activation of complement played a significant role in promoting the sensitiveness of necroptosis (Shi et al., 2015). Overall, these findings provide new insights into the role of necroptosis in DN.

Subsequently, via the selection of ML and WGCNA, a total of 8 significant NRDEGs were identified, including CFLAR, FMR1, GSDMD, IKBKB, MAP3K7, NFKBIA, PTGES3, and SFTPA1. Particularly, several significant NRDEGs have been confirmed that regulated the immune landscape and inflammatory response via necroptosis (Yu et al., 2022; Chen et al., 2023). CFLAR is a crucial regulator in apoptosis, autophagy, and necroptosis [23,392,074]. He et al. indicated that cells, especially T cells, with the abnormal expression of CFLAR suffered form severe apoptosis and necroptosis (He and He, 2013). And FMRP (Protein of FMR1) binds RIPK1 mRNA, suggesting that FMRP acts as a regulator of necroptosis pathway through the surveillance of RIPK1 mRNA metabolism in colorectal cancer (Zhuang et al., 2020; Di Grazia et al., 2021). Meanwhile, in mitochondria, GSDMD promotes the release of ROS to induce necroptosis (Weindel et al., 2022). And abundant studies have indicated that GSDMD induces the occurrence, development and inflammation of DN via pyroptosis (Cheng et al., 2021; Zuo et al., 2021). Our research focused for the first time on the relationship between GSDMD and necrotic apoptosis in DN, which further emphasized the role of GSDMD in PCD and DN. Kondylis et al. reviewed the characteristic of IKK, NF-κB and RIPK1 signaling in necroptosis, tissue homeostasis and inflammation, which provided a powerful basis for the obtaining of IKBKB, NFKBIA and MAP3K7 as significant NRDEGs of DN in our research (Kondylis et al., 2017). IKBKB can alleviate the neuron injury in Alzheimer’s Disease via regulating autophagy and RIPK1-Mediated necroptosis [35,083,662]. The phosphorylation mediated by MAP3K7 (TAK1) can regulate the activation of RIPK1 to dictate the apoptosis and necroptosis [28,842,570]. Similarly, the phosphorylation of NFKBIA was correlated with the necroptosis in breast cancer cells [34,030,642]. And the above 3 NRDEGs have been regarded as inflammatory regulators in DN in the previous studies (Zhang et al., 2007; Oguiza et al., 2015). As the co-chaperone of HSP90, PTGES3 (P23) has been found can activate the RIPK3/MLKL during the necroptosis in acute respiratory distress syndrome [32,072,232]. Moreover, the mutation of homozygous SFTPA1 drives the necroptosis of type II alveolar epithelial cells in idiopathic pulmonary fibrosis [31,601,679]. Overall, our 8 significant NRDEGs all can be regarded that play a direct or potential role in necroptosis in various diseases. And in DN, our study first indicated the characteristic of these 8 genes between DN and necroptosis, which may provide some novel insights in personalized therapy strategies for DN.

Based on the 8 significant NRDEGs, we identified two different necroptosis-related DN subtypes with different necroptosis score. Meanwhile, the above subtypes exhibited diversified immune infiltration and inflammatory responses with different ESTIMATE score, CYT score and expression level of ICKs, which further demonstrated the regulatory effect of necroptosis on immune microenvironment and inflammation in DN (Erekat, 2022). In addition, the single-cell analysis revealed that in podocytes, almost all the 8 significant NRDEGs exhibited remarkable expression difference between CN and DN, which implied that necroptosis may play a critical role in the injury of podocytes of DN. Meanwhile, in other renal tubular cells, epithelial cells and leukocytes, several NRDEGs also exhibited difference, further emphasizing the role of necroptosis in the damage of renal parenchymal of DN patients. Finally, a necroptosis-mediated model was constructed with the significant diagnostic efficiency in both training set and validation sets, whose AUC was 0.97, exhibiting better diagnostic efficiency than individual significant NRDEGs.

Overall, in our present work, we integrated the transcriptome data and single-cell data to excavate the crucial characteristic of necroptosis in DN and identified 8 significant NRGs for the diagnosis and subtyping of DN. However, due to regulatory and ethical concerns, collecting the glomerulus samples of human was excessively difficult and the expression level of these NRGs were only validated in the animal level via RT-qPCR. Notably, research about the relationship between the eight significant NRDEGs and the kidney functions was still rare, our research firstly indicated the potential correlated relationship between the eight significant NRDEGs and the kidney function of DN. Then, based on the landscape of necroptosis-regulators in DN, we identified two DN subtypes with diversified immune microenvironment and inflammatory response, which may provide new insights in exploiting personalized therapy strategies for DN patients. And regretfully, the detailed mechanism of the 8 NRGs and necroptosis in DN was not fully discussed and explored in our work, which was worthy to experimented in the future. Finally, although the necroptosis-mediated diagnostic model exhibited superior efficiency, the validation of the model is temporarily limited to bioinformatics and no further experiments have been conducted for verification.

## Conclusion

Research about necroptosis in DN was still rare, especially via bioinformatic analysis. Our work exhibited the landscape of necroptosis in DN and ensured 8 NRGs with significant expression difference in the glomerulus tissues between DN and CN via bioinformatics and validation experiments. In addition, two necroptosis-related subtypes with different inflammatory response and immune microevironment were identified, according to which we calculated the necroptosis score to evaluate the immune landscape and inflammatory response of DN patients. Finally, we developed a necroptosis-mediated diagnostic model to accurately diagnose DN patients. In conclusion, our work demonstrated an accurate and novel model aiming to contribute in the field of precision diagnosis and personalized therapy of DN patients, which further consummate the relationship between DN and necroptosis and may become a shining novel star in further research.

## Data Availability

Publicly available datasets were analyzed in this study. This data can be found here: The original data used in this project can be downloaded in the public database GEO (https://www.ncbi.nlm.nih.gov/geo/). Accession Numbers: GSE96804, GSE30528 and GSE30122.

## Supplementary material

SUPPLEMENTARY FIGURE S1 The biological background and functional enrichment analysis for the two different necroptosis-related subtypes. **(A)** The proportion of each pathway in the results of ClueGO enrichment analysis. **(B-D)** The significant modules identified from the gene-pathway interaction network with the corresponding score, nodes and edges. **(E, F)** The results of the **(E)** KEGG and **(F)** Reactome enrichment analysis. **(G-I)** The pathways identified via the GSEA algorithm with normalized p-value < 0.05.

SUPPLEMENTARY FIGURE S2 The kidney operated from our mice model, **(A)** the CN group, **(B)** the DN group.
