# Supplementary material for: Identification of necroptosis-related features in diabetic nephropathy and analysis of their immune microenvironent and inflammatory response
Source: Front Cell Dev Biol. 2023 Nov 7;11:1271145. doi: 10.3389/fcell.2023.1271145 (PMC10661379; doi:10.3389/fcell.2023.1271145)
Supplement: Supplementary file 1 [file DataSheet1.ZIP › Table S2.docx]

| **Gene** | **Primer** | **Sequence (5'-3')** |
| --- | --- | --- |
| CFLAR | Forward | GCTCCAGAATGGGCGAAGTAA |
|  | Reverse | ACGGATGTGCGGAGGTAAAAA |
| FMR1 | Forward | CAATGGCGCTTTCTACAAGGC |
|  | Reverse | TCTGGTTGCCAGTTGTTTTCA |
| GSDMD | Forward | CCATCGGCCTTTGAGAAAGTG |
|  | Reverse | ACACATGAATAACGGGGTTTCC |
| IKBKB | Forward | ACAGCCAGGAGATGGTACG |
|  | Reverse | CAGGGTGACTGAGTCGAGAC |
| MAP3K7 | Forward | CGGATGAGCCGTTACAGTATC |
|  | Reverse | ACTCCAAGCGTTTAATAGTGTCG |
| NFKBIA | Forward | TGAAGGACGAGGAGTACGAGC |
|  | Reverse | TTCGTGGATGATTGCCAAGTG |
| PTGES3 | Forward | TGTTTGCGAAAAGGAGAATCCG |
|  | Reverse | CCATGTGATCCATCATCTCAGAG |
| SFTPA1 | Forward | GAGGAGCTTCAGACTGCACTC |
|  | Reverse | AGACTTTATCCCCCACTGACAG |
| GPADH | Forward | GGAAGCTTGTCATCAATGGAAATC |
|  | Reverse | TGATGACCCTTTTGGCTCCC |

**Table S2.** The primer pairs utilized in Real-Time Quantitative PCR.
